# Cyclin E/CDK2: DNA Replication, Replication Stress and Genomic Instability

**DOI:** 10.3389/fcell.2021.774845

**Published:** 2021-11-24

**Authors:** Rafaela Fagundes, Leonardo K. Teixeira

**Affiliations:** Group of Cell Cycle Control, Program of Immunology and Tumor Biology, Brazilian National Cancer Institute (INCA), Rio de Janeiro, Brazil

**Keywords:** cyclin E, CCNE, CDK2, DNA replication, replication stress, genomic instability, cell cycle, cancer

## Abstract

DNA replication must be precisely controlled in order to maintain genome stability. Transition through cell cycle phases is regulated by a family of Cyclin-Dependent Kinases (CDKs) in association with respective cyclin regulatory subunits. In normal cell cycles, E-type cyclins (Cyclin E1 and Cyclin E2, *CCNE1* and *CCNE2* genes) associate with CDK2 to promote G1/S transition. Cyclin E/CDK2 complex mostly controls cell cycle progression and DNA replication through phosphorylation of specific substrates. Oncogenic activation of Cyclin E/CDK2 complex impairs normal DNA replication, causing replication stress and DNA damage. As a consequence, Cyclin E/CDK2-induced replication stress leads to genomic instability and contributes to human carcinogenesis. In this review, we focus on the main functions of Cyclin E/CDK2 complex in normal DNA replication and the molecular mechanisms by which oncogenic activation of Cyclin E/CDK2 causes replication stress and genomic instability in human cancer.

## Introduction

Cellular proliferation is controlled by an intricate network of proteins that dictate the order and timing of cell cycle events. Progression through cell cycle phases is regulated by a family of Cyclin-Dependent Kinases (CDKs), which associate with respective Cyclin regulatory subunits. Oscillations in Cyclin levels determine fluctuations in CDK activity, which ultimately control cell cycle phase transitions (Malumbres and Barbacid, 2009; Matthews et al., 2021). In normal mammalian cells, expression of E-type cyclins, named Cyclin E1 and Cyclin E2, is reached as a consequence of RB inactivation and E2F transcription factor release, which is initially caused by Cyclin D/CDK4-6 activation upon mitogenic stimulation during G1. E2F-mediated Cyclin E transcription is followed by Cyclin E protein accumulation that peaks at the G1/S transition, when Cyclin E binds and activates CDK2 to promote S phase entry and progression. Cyclin E/CDK2 complex then phosphorylates numerous substrates to control essential cellular processes, such as progression through the restriction point (R point), initiation of DNA replication, and regulation of histone biosynthesis among others. By the end of S phase, Cyclin E protein levels are completely degraded by the SCF^FBW7^ ubiquitin ligase complex, thus eliminating Cyclin E/CDK2 activity up to the subsequent G1 phase (Hwang and Clurman, 2005; Chu et al., 2021).

Oncogenic activation of Cyclin E/CDK2 complex is frequently observed in human cancers and may be achieved by different genetic events, such as amplification of Cyclin E genes (*CCNE1* or *CCNE2*), disruption of the RB/E2F pathway (leading to increased Cyclin E transcription), and mutation of *FBXW7* ubiquitin ligase (causing accumulation of Cyclin E protein). In fact, high levels of Cyclin E protein and increased CDK2 kinase activity are both independently associated with poor prognosis, reduced survival, and therapy resistance in cancer patients (Hwang and Clurman, 2005). Under the cell cycle perspective, oncogenic activation of Cyclin E/CDK2 complex has been largely demonstrated to impair DNA replication, causing DNA replication stress, which may be defined as the slowing or stalling of replication fork progression during DNA synthesis upon different insults. Hyperactivation of Cyclin E/CDK2 complex directly interferes with DNA replication through several mechanisms, leading to DNA double strand breaks (DSBs) and genomic instability. In fact, specific targeting of oncogenic Cyclin E/CDK2 complex has been proposed as a promising therapy against cancer (Tadesse et al., 2020; Suski et al., 2021).

Regulation of E-type cyclins and the effects of Cyclin E/CDK2 complex in normal physiology and disease states have been extensively reviewed in the literature (Hwang and Clurman, 2005; Caldon and Musgrove, 2010; Siu et al., 2012; Chu et al., 2021). In this review, we focus on the role of Cyclin E/CDK2 complex in DNA replication and the molecular mechanisms by which hyperactivation of Cyclin E/CDK2 complex causes DNA replication stress and genomic instability in human cancer.

## Cyclin E: Structure and Function

**FIGURE 1:**
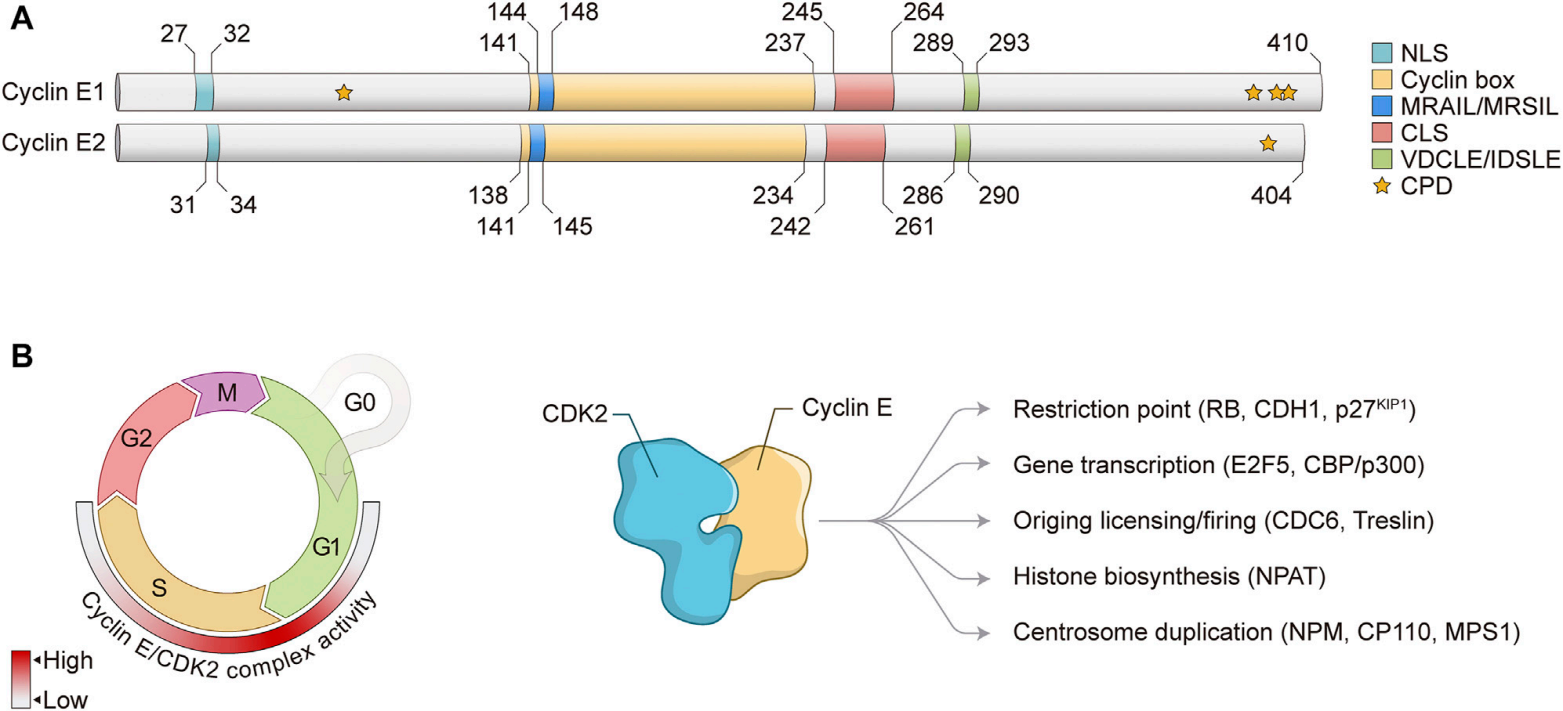
Cyclin E structure and function in the cell cycle. **(A)** Schematic representation of Cyclin E1 and Cyclin E2 protein structures. Numbers represent amino acid positions. NLS, nuclear localization signal; CLS, centrosome localization signal; CPD, CDC phosphodegrons. **(B)** Left: Cyclin E/CDK2 complex controls G1/S phase transition and S phase progression. Heat map indicates Cyclin E/CDK2 complex activity through the cell cycle. Right: Cyclin E/CDK2 complex regulates different cell cycle-related events and substrates. RB, Retinoblastoma; CDH1, CDC20 homolog 1; KIP1, CDK inhibitory protein 1; E2F, E2 promoter-binding factor; CBP, CREB-binding protein; CDC, Cell division cycle; NPAT, Nuclear protein, coactivator of histone transcription; NPM, Nucleophosmin; CP110, Centriolar coiled-coil protein 110; MPS1, Monopolar spindle 1.

Cyclin E1 was the first member of the E-type cyclin family to be identified (Koff et al., 1991; Lew et al., 1991). In humans, *CCNE1* gene localizes at 19q12 region and encodes for a full-length protein of 410 amino acids (Figure 1A). Several splice variants and protein isoforms have also been described for *CCNE1* gene. Cyclin E1 protein has been shown to bind and activate CDK2 kinase, creating an active complex that is responsible for S phase entry and progression (Ohtsubo and Roberts, 1993; Resnitzky et al., 1994). In accordance with its role in S phase promotion, the highest levels of Cyclin E1 mRNA are observed during G1/S phase transition, coinciding with maximum activity of Cyclin E1/CDK2 complex (Dulić et al., 1992; Koff et al., 1992). Cyclin E2 was the second member of the E-type cyclin family to be described in humans (Lauper et al., 1998; Zariwala et al., 1998; Gudas et al., 1999). *CCNE2* gene is localized at 8q22.1 region and encodes for a full-length protein of 404 amino acids (Figure 1A). Like Cyclin E1, Cyclin E2 protein also binds and activates CDK2, forming an active kinase complex whose activity also peaks during G1/S phase transition (Lauper et al., 1998; Zariwala et al., 1998; Gudas et al., 1999). Cyclin E1 and E2 proteins show approximately 50% of overall sequence identity and are assumed to be functionally redundant. However, several reports have indicated distinct regulation and functions for Cyclin E1 and E2 proteins (Caldon and Musgrove, 2010).

Cyclin E is a nuclear protein that mainly exerts its regulatory functions through interaction with and activation of CDK2 to induce phosphorylation of target proteins. Of note, it has been shown that Cyclin E1 can also bind and activate CDK1 *in vivo* (Aleem et al., 2005), however CDK2 is the main binding partner of Cyclin E. Cyclin E interacts with CDK2 through its Cyclin box with the PSTAIRE helix on CDK2, leading to conformational changes on the CDK2 T loop (Honda et al., 2005) (Figure 1A). Exposure of CDK2 catalytic site allows for activating phosphorylation of CDK2 Thr160 by CDK activating kinase (CAK). Interaction between Cyclin E/CDK2 complex with substrates is mediated by two distinct domains found on Cyclin E: MRAIL and VDCLE (Figure 1A). The MRAIL domain is localized at the N-terminal region of the Cyclin box and mediates binding to RLX-containing proteins, such as RB, p27^KIP1^, and CDC6 (Adams et al., 1996; Chen et al., 1996; Schulman et al., 1998; Furstenthal et al., 2001). The VDCLE domain is found on Cyclin E C-terminal portion and regulates the interaction with the pocket protein family members RB1, p107, and p130 (Dowdy et al., 1993; Kelly et al., 1998). Of note, similar sequences are observed in Cyclin E2 protein: MRSIL and IDSLE, respectively (Figure 1A). Two additional domains are observed on Cyclin E1 and E2 proteins: nuclear localization signal (NLS) and centrosome localization signal (CLS) (Figure 1A). The N-terminal NLS potentially contributes to Cyclin E nuclear localization, however it is clear that other mechanisms also regulate Cyclin E nuclear accumulation in human cells (Jackman et al., 2002; Moore et al., 2002). The CLS targets Cyclin E to the centrosomes, where it is essential for Cyclin E/CDK2-mediated centrosome duplication (Matsumoto and Maller, 2004). Furthermore, E-type cyclins also show CDC phosphodegrons (CPDs) at the N- and C-terminus, which are represented by Serine and Threonine (S/T) residues that are initially phosphorylated by certain kinases and later recognized by the SCF^FBW7^ ubiquitin ligase complex and directed to proteasomal degradation (Clurman et al., 1996; Won and Reed, 1996) (Figure 1A). Importantly, Cyclin E/CDK2 activity is negatively regulated by the KIP/CIP family of CDK inhibitors, p27^KIP1^ and p21^CIP1^, which prevent CDK2 activation by CAK phosphorylation and also inhibit Cyclin E/CDK2 complex interaction with substrates (Sherr and Roberts, 2004).

Cyclin E levels are tightly regulated throughout the cell cycle by a timely combination of gene expression and protein degradation. During G1, mitogenic stimulation induces Cyclin D accumulation, which together with CDK4/6 phosphorylates and inactivates RB, releasing activating E2F1-3 transcription factors to induce Cyclin E transcription (Ohtani et al., 1995; Geng et al., 1996). Upon S phase progression, Cyclin E transcription is repressed through the assembly of inhibitory proteins to Cyclin E promoter, including E2F6 and E2F7 transcriptional repressors (Giangrande et al., 2004; Westendorp et al., 2012). Apart from transcriptional repression, protein degradation is mostly responsible for progressive decrease of Cyclin E levels through S phase. Degradation of Cyclin E1 protein is mediated by phosphorylation of several S/T residues (CPDs) observed within the N-terminal (T77, also known as T62) and C-terminal regions (S387, T395, and S399, also known as S372, T380, and S384) of full-length Cyclin E1 protein (Clurman et al., 1996; Won and Reed, 1996; Welcker et al., 2003; Ye et al., 2004) (Figure 1A). Phosphorylation of Cyclin E1 CPDs may be achieved by either GSK3 or CDK2 autophosphorylation. Phosphorylated Cyclin E1 is then mostly recognized by the SCF^FBW7^ ubiquitin ligase complex and subsequently marked for ubiquitin-mediated degradation via proteasome (Koepp et al., 2001; Strohmaier et al., 2001). On the other hand, Cyclin E2 protein degradation has not been fully investigated yet, but it is assumed that its proteolysis is similar to Cyclin E1. Cyclin E2 protein C-terminal residue T392 is conserved to Cyclin E1 protein residue T395 and its phosphorylation is also required for protein degradation (Lauper et al., 1998) (Figure 1A). Of note, Cyclin E protein may be cleaved by intracellular proteolytic processing, generating low-molecular weight Cyclin E (LMW-E) isoforms. These LMW-E lack the N-terminal NLS, accumulate in the cytoplasm, and have been shown to present increased affinity for CDK2 and resistance to CDK inhibitors p21^CIP1^ and p27^KIP1^ (Caruso et al., 2018).

In normal conditions, Cyclin E/CDK2 complex controls several critical biological functions (Figure 1B). During G1/S transition, Cyclin E/CDK2 phosphorylates and inactivates RB protein, leading to release of E2F transcription factors and a positive feedback loop for Cyclin E transcription (Harbour and Dean, 2000). Through inactivation of RB and release of activating E2Fs, Cyclin E/CDK2 complex activity induces the expression of a variety of genes that are essential for S phase entry and progression, such as cell division cycle 6 (*CDC6*), chromatin licensing and DNA replication factor 1 (*CDT1*) and members of the minichromosome maintenance (MCM) complex, all three components of pre-replication complex (pre-RC); A-type cyclins; and EMI1, inhibitor of the APC/C^CDC20^ complex (Ishida et al., 2001; Hsu et al., 2002; Polager et al., 2002). Besides phosphorylation of canonical RB protein, Cyclin E/CDK2 complex also phosphorylates other critical targets for cell cycle progression, including p27^KIP1^ CDK inhibitor; E2F5 and CBP/p300 transcription factors; NPAT and HIRA, proteins involved in histone biosynthesis; and NPM, CP110 and MPS1, all involved in centrosome duplication (Figure 1B) (Sheaff et al., 1997; Ait-Si-Ali et al., 1998; Ma et al., 2000; Morris et al., 2000; Okuda et al., 2000; Zhao et al., 2000; Fisk and Winey, 2001; Hall et al., 2001; Tokuyama et al., 2001; Chen et al., 2002). High-throughput proteomic screening approaches have been performed to determine the profile of Cyclin E1 interactome, as well as to identify novel CDK2 substrates (Pagliuca et al., 2011; Odajima et al., 2016; Chi et al., 2020). These studies have provided powerful resources for the investigation of Cyclin E/CDK2 regulatory network and function. Furthermore, as CDK2 may be activated by Cyclin E and Cyclin A, these findings are instrumental to the analysis of similarities and differences between Cyclin E- and Cyclin A-associated CDK2 activity, which are essential for timely progression of the cell cycle (Pagliuca et al., 2011; Chi et al., 2020). For the purpose of this review, we will focus on the functions of Cyclin E/CDK2 complex in normal and aberrant DNA replication.

## Cyclin E/CDK2 Complex in DNA Replication

Eukaryotic cells must ensure accurate chromosome replication in order to maintain genome stability. DNA replication is a multi-step process that is characterized by the chronological assembly of different protein complexes onto DNA replication origins (ORIs), followed by replisome activation and subsequent DNA synthesis. From origin licensing to replication completion, all necessary steps are tightly regulated to permit appropriate genome replication. Alterations that compromise the function of key proteins involved in DNA replication can lead to progressive accumulation of errors in the DNA molecule and genomic instability (Masai et al., 2010; Bellelli and Boulton, 2021).

**FIGURE 2:**
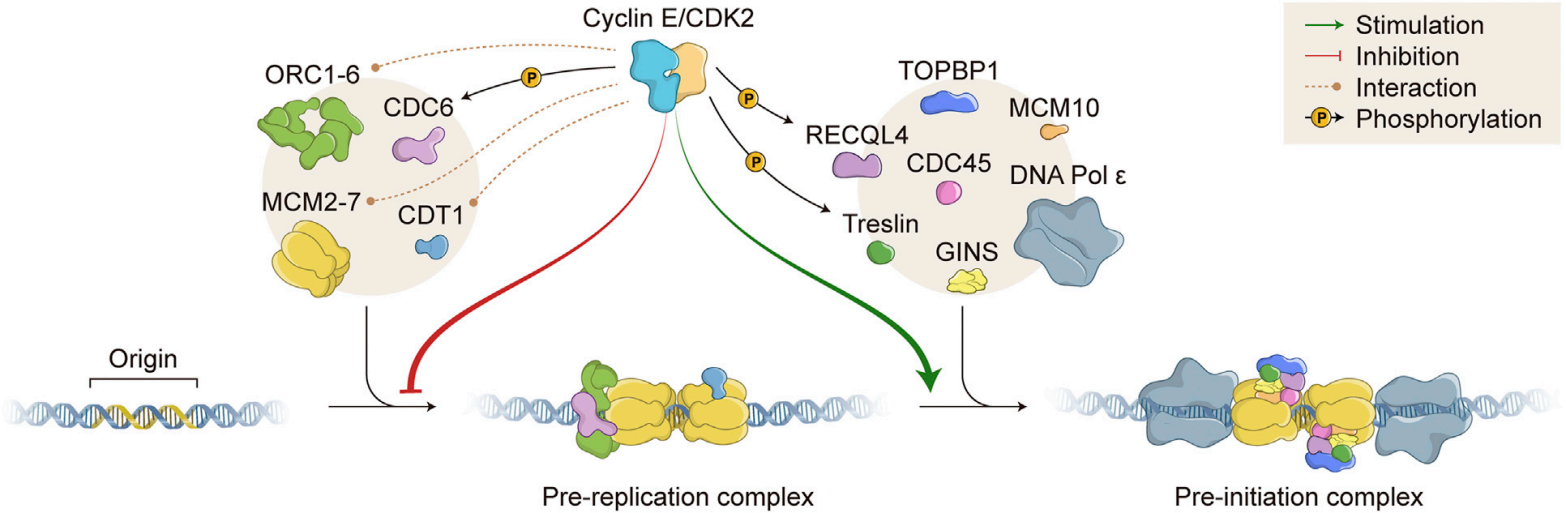
Role of Cyclin E/CDK2 in DNA replication. Cyclin E/CDK2 complex directly interacts with (orange dashed lines) and phosphorylates (black arrows; P, phosphate) several proteins that are essential for DNA replication. Cyclin E/CDK2 activity inhibits pre-replication complex (pre-RC) formation (red arrow) and stimulates pre-initiation complex (pre-IC) formation (green arrow). DNA replication origins (Origin) are recognized by different proteins, which are sequentially recruited to form pre-RC, including ORC1-6 and MCM2-7 complexes (left beige circle). Subsequently, many other proteins are assembled onto DNA replication origins to form pre-IC, including TOPBP1/RECQL4/Treslin and CDC45/GINS (right beige circle). DNA molecule (blue double strand); ORC, Origin recognition complex; CDC, Cell division cycle; MCM, Minichromosome maintenance; CDT1, Chromatin licensing and DNA replication factor 1; CDK, Cyclin dependent kinase; TOPBP1, DNA topoisomerase II binding protein 1; RECQL4, RecQ like helicase 4; GINS, Go-ich-ni-san. Numerous replisome proteins are omitted for simplicity.

In order to accomplish one single round of DNA replication per cell cycle, two fundamental steps that precede DNA synthesis have to be executed in a temporally separated manner: origin licensing and origin firing. Origin licensing takes place during late mitosis and early G1 phase, when cells possess low levels of CDK activity, and involves the assembly of pre-RC onto ORIs. Origin licensing starts with the binding of origin recognition complex subunits 1–6 (ORC1-6) and CDC6 to ORIs, followed by recruitment of CDT1 and MCM2-7 helicase complex, completing pre-RC assembly (Figure 2). At this point, ORIs are inactive, though primed for later activation in the cell cycle (Masai et al., 2010; Siddiqui et al., 2013; Fragkos et al., 2015). Origin activation takes place during G1/S transition and requires the recruitment of additional proteins to generate pre-initiation complex (pre-IC) (Figure 2). Unlike origin licensing, pre-IC formation requires high kinase activity that results from the combined action of two S phase kinases: CDK2 and CDC7 (also known as DDK, DBF4-dependent kinase), which associate with regulatory subunits Cyclin E/A and DBF4, respectively. Together, these kinases phosphorylate several replication factors and facilitate protein recruitment to ORIs, including the recruitment of CDC45 and GINS to allow formation of the CMG helicase complex (CDC45-MCM-GINS). Helicase activation and subsequent DNA unwinding leads to the recruitment of other additional proteins, including DNA polymerases, and eventually origin firing (Masai et al., 2010; Tanaka and Araki, 2013; Fragkos et al., 2015; Burgers and Kunkel, 2017).

Cyclin E/CDK2 complex has essential and opposing roles in DNA replication (Figure 2). As mentioned before, origin licensing occurs when cells experience low CDK environments. Accordingly, hyperactivation of Cyclin E1/CDK2 complex impairs MCM loading and prevents pre-RC assembly, causing DNA replication stress (see below). However, under certain circumstances, normal levels of Cyclin E/CDK2 complex are essential to promote origin licensing. It has been shown that cells deficient for Cyclin E1/E2 or depleted of CDK2 activity are not able to re-enter the cell cycle from quiescence due to failure in MCM loading (Geng et al., 2003; Chuang et al., 2009). In fact, during cell cycle re-entry from quiescence, Cyclin E/CDK2 complex activity is required for accumulation of *CDC6* and *CDC7* mRNA levels, both of which are necessary for MCM loading (Chuang et al., 2009). Cyclin E/CDK2 complex is also able to directly interact with pre-RC components and form a ternary complex with ORC1 and CDC6 proteins, both acting as receptors for Cyclin E/CDK2 binding onto chromatin during origin licensing (Furstenthal et al., 2001; Hossain and Stillman, 2016; Hossain et al., 2021) (Figure 2). In agreement, it has been shown that Cyclin E/CDK2 complex and CDC6 protein work synergistically to promote pre-RC assembly and abrogation of Cyclin E-CDC6 interaction leads to failure in DNA replication (Furstenthal et al., 2001; Cook et al., 2002; Coverley et al., 2002). Cyclin E1 has been also shown to physically interact with CDT1 and members of the MCM complex, possibly further facilitating MCM loading onto ORIs (Geng et al., 2007; Odajima et al., 2016) (Figure 2). In terms of phosphorylation of pre-RC components, Cyclin E1/CDK2 complex has been shown to directly phosphorylate CDC6 in human cells, protecting CDC6 from APC/C-mediated ubiquitylation and proteolysis. Cyclin E/CDK2-mediated CDC6 phosphorylation potentiates MCM loading onto chromatin and allows for DNA replication (Mailand and Diffley, 2005) (Figure 2). Additional reports have suggested that Cyclin E/CDK2 complex is able to phosphorylate other proteins that constitute pre-RCs, such as CDT1 and MCM complex components, possibly interfering with chromatin loading or protein-protein interaction. However, further definitive work is still necessary to clearly demonstrate and elucidate the effects of Cyclin E/CDK2-mediated phosphorylation of other pre-RC components in human cells.

Apart from regulating pre-RC assembly, Cyclin E/CDK2 complex exerts a positive role during pre-IC formation, which occurs upon high kinase activity (Figure 2). It has been shown that Cyclin E/CDK2 directly phosphorylates Treslin (also known as TICRR, TOPBP1 interacting checkpoint and replication regulator), promoting its interaction with TOPBP1 (DNA topoisomerase II binding protein 1) and facilitating recruitment of CDC45, GINS, and DNA polymerases onto chromatin (Figure 2). Indeed, interaction of Treslin-TOPBP1 induced by Cyclin E/CDK2 phosphorylation is essential for initiation of DNA replication *in vivo* (Kumagai et al., 2010; Boos et al., 2011; Kumagai et al., 2011; Sansam et al., 2015). Besides Treslin, Cyclin E/CDK2 complex also phosphorylates RECQL4 (RecQ like helicase 4), increasing its helicase activity (Figure 2). However, it is still not clear whether Cyclin E/CDK2-dependent RECQL4 phosphorylation favours its chromatin binding and/or pre-IC formation (Lu et al., 2017). Consistently, Sld2 and Sld3, the yeast counterparts of human RECQL4 and Treslin, respectively, have been shown to bind Dpb11 (the yeast counterpart of human TOPBP1) upon CDK phosphorylation. CDK-dependent Sld2 and Sld3 phosphorylations in yeast are essential for the initiation of DNA replication (Tanaka et al., 2007; Zegerman and Diffley, 2007).

## Oncogenic Activation of Cyclin E/CDK2 Mediates Replication Stress

As discussed above, Cyclin E/CDK2 complex plays a central role in controlling normal DNA replication. Therefore, it is expected that oncogenic activation of Cyclin E/CDK2 interferes with DNA synthesis and causes replication stress. Indeed, it has been shown that Cyclin E1 overexpression impairs replication fork progression, leading to premature termination of replication forks, fork collapse, and DSBs (Bartkova et al., 2005, 2006). Importantly, Cyclin E-mediated replication stress is directly associated with increased CDK2 activity, as a hyperactive *CDK2* allele is sufficient to impair replication fork progression and cause DNA damage (Hughes et al., 2013). In normal cells, aberrant activation of Cyclin E1/CDK2 complex induces the replication stress response (RSR), leading to cell cycle arrest, cell death, and senescence. This is an essential mechanism to prevent tumor progression in normal tissues. However, oncogenic activation of Cyclin E1/CDK2, associated with disruption of the RSR pathway, allows for increased cell proliferation in the presence of sustained replication stress, contributing to genomic instability in early steps of human tumorigenesis (Bartkova et al., 2005, 2006; Teixeira and Reed, 2017).

**FIGURE 3:**
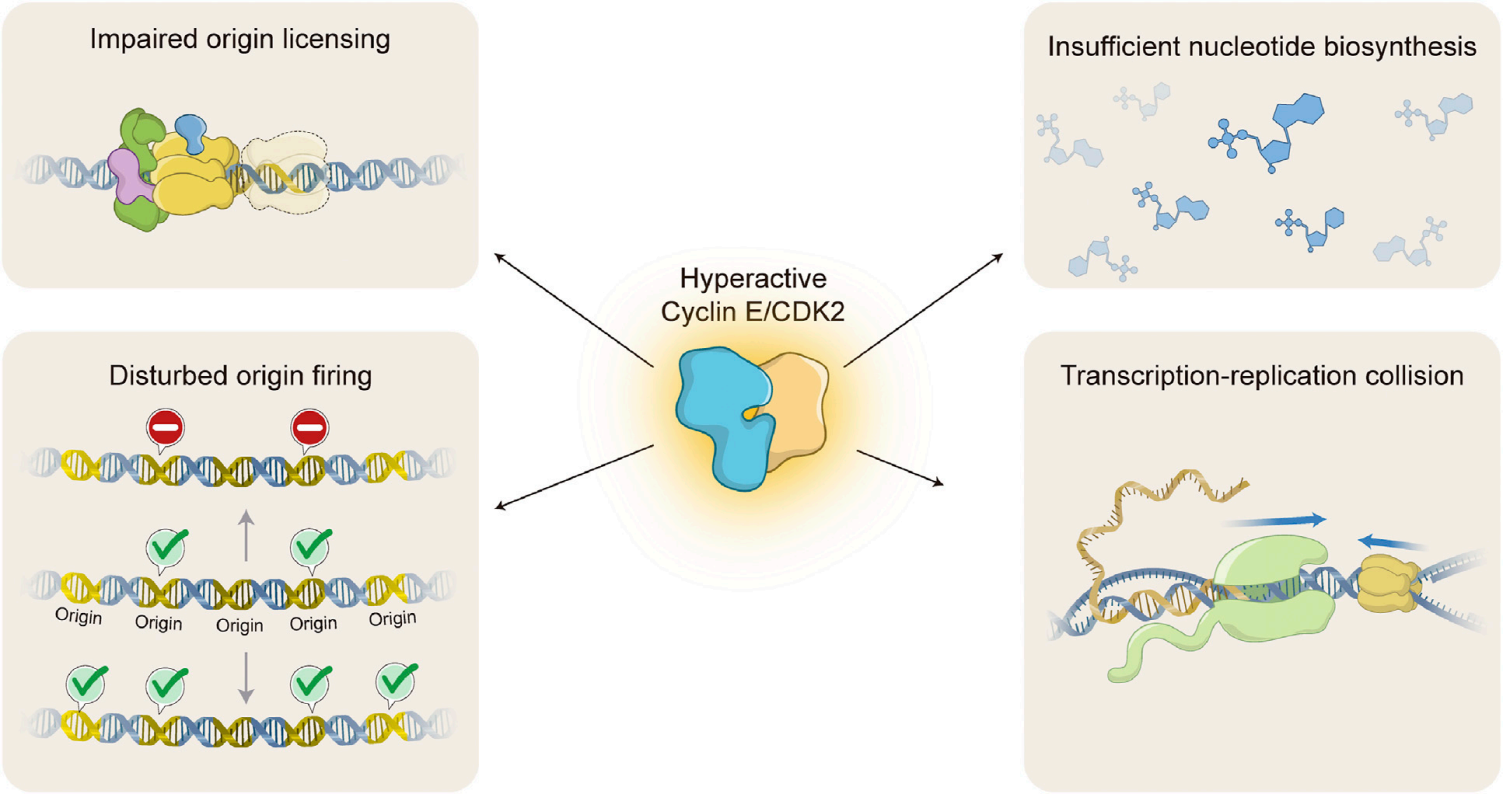
Molecular mechanisms of Cyclin E/CDK2-induced replication stress. Oncogenic activation of Cyclin E/CDK2 interferes with DNA replication through several different molecular mechanisms, causing DNA replication stress. Hyperactive Cyclin E/CDK2 complex impairs origin licensing by interference with pre-replication complex (pre-RC) loading onto chromatin **(upper left)** and origin firing by either decreasing or increasing activation of DNA replication origins (**lower left**; upper or lower DNA molecule, respectively). Hyperactive Cyclin E/CDK2 complex also leads to insufficient nucleotide pools by forcing cell hyperproliferation without accompanying nucleotide biosynthesis **(upper right)**. Finally, hyperactive Cyclin E/CDK2 complex causes transcription-replication collisions due to imbalances in DNA replication and gene transcription processes **(lower right)**. Upper left: DNA molecule (blue double strand); ORC complex (green); CDC6 (purple); MCM complex (yellow); CDT1 (blue); Faded MCM complex represents impaired origin licensing. Lower left: DNA molecule (blue double strand); DNA replication origins (Origin, yellow double strand); Activated origin firing (green check); Impaired origin firing (stop signal). Upper right: chemical structures represent different nucleotides (blue). Lower right: DNA molecule (blue double strand); messenger RNA (yellow single strand); RNA polymerase (light green); MCM complex (yellow); R-loop (RNA-DNA hybrid molecule). Blue arrows indicate direction of transcription and replication progression. Replisome is omitted for simplicity.

Oncogenic activation of Cyclin E/CDK2 complex is able to mediate replication stress by several different molecular mechanisms (Figure 3). One of the primary mechanisms is interference with origin licensing (Figure 3, upper left). As discussed above, pre-RC assembly onto chromatin occurs during late mitosis and early G1, when cells experience low CDK environments (Masai et al., 2010; McIntosh and Blow, 2012). Unscheduled CDK activity during these cell cycle stages is likely to compromise origin licensing. Indeed, it has been demonstrated that overexpression of yeast G1 cyclin Cln2 inhibits pre-RC assembly and leads to gross chromosomal rearrangements (Tanaka and Diffley, 2002). Consistently, high levels of Cyclin E1 at the M/G1 boundary, accompanied by hyperactive CDK2 activity, impair loading of specific MCM helicase components onto chromatin in mammalian cells (Ekholm-Reed et al., 2004). One possible explanation is that oncogenic Cyclin E1/CDK2 activity forces rapid progression through G1 and premature entry into S phase with insufficient pre-RC formation. However, the molecular mechanism by which hyperactive Cyclin E/CDK2 complex interferes with MCM loading and/or distribution onto chromatin is still not understood.

Cyclin E/CDK2-induced replication stress may be caused not only by interference with origin licensing, but also with origin firing (Figure 3, lower left). Indeed, Cyclin E1 overexpression has been shown to decrease origin firing (Liberal et al., 2012), consistent with the idea that cells experiencing oncogene-induced replication stress trigger the intra-S-phase checkpoint to prevent new origin activation (Gaillard et al., 2015). On the other hand, in agreement with a positive role of Cyclin E/CDK2 complex in origin activation, hyperactivation of Cyclin E1/CDK2 complex has been shown to aberrantly induce origin firing (Hughes et al., 2013; Jones et al., 2013). High levels of Cyclin E1 are able to interfere with time and location of origin activation, inducing premature, novel origin firing in intragenic regions (Macheret and Halazonetis, 2018). Again, the molecular mechanisms for disturbed origin firing upon oncogenic Cyclin E/CDK2 activation remain to be determined.

Another important mechanism by which Cyclin E/CDK2 hyperactivation mediates replication stress is interference with nucleotide pools (Figure 3, upper right). Precise regulation of nucleotide metabolism and biosynthesis is essential for execution of numerous biological processes, including DNA replication and RNA production (Lane and Fan, 2015). Reduction in nucleotide availability directly interferes with DNA replication dynamics (Anglana et al., 2003). It has been shown that Cyclin E1 overexpression induces aberrant activation of the RB/E2F pathway without accompanying increase in nucleotide biosynthesis (Bester et al., 2011). As a consequence, cells are enforced to proliferate with insufficient levels of nucleotides, leading to decreased progression of replication forks and induction of DSBs. Interestingly, supplementation with exogenous nucleosides is able to attenuate Cyclin E1-mediated replication stress and DNA damage (Bester et al., 2011).

Transcription-replication collisions represent another source of replication stress that may be induced by hyperactive Cyclin E/CDK2 complex (Figure 3, lower right). Precise regulation of Cyclin E/CDK2 activity is not only essential for normal DNA replication, but also for appropriate RNA production as it represents a critical step in RB inactivation and subsequent E2F-mediated transcription activation (Harbour and Dean, 2000). Initially, it has been shown that inhibition of either replication initiation or transcription elongation are able to alleviate replication stress induced by Cyclin E1 overexpression (Jones et al., 2013). Later on, it has been demonstrated that high levels of Cyclin E1 induce inappropriate origin firing within intragenic, coding sequences, leading to transcription-replication conflicts, replication fork collapse, DSBs, and chromosomal aberrations (Macheret and Halazonetis, 2018). Transcription-replication collisions may cause DNA topological tension and formation of persistent R-loops (RNA-DNA hybrid structures) (Helmrich et al., 2013). Indeed, it has been shown that Cyclin E1 overexpression induces accumulation of aberrant DNA replication intermediates, such as reversed replication forks (Neelsen et al., 2013). Altogether, hyperactivation of Cyclin E/CDK2 complex clearly interferes with normal DNA replication, causing replication stress and contributing to genomic instability.

## Cyclin E/CDK2-Induced Replication Stress Causes Genomic Instability in Human Cancers

Genomic instability is a hallmark of cancer and is defined as an increased frequency of genetic alterations during cell divisions. It may be caused by cell extrinsic or intrinsic genotoxic insults, and is observed in human cancers under various forms, such as whole chromosome gains and/or losses, focal copy number alterations, chromosomal rearrangements, and clustered base-pair mutations (Negrini et al., 2010; Aguilera and García-Muse, 2013). Oncogene activation, another hallmark of cancer, may interfere with normal cell proliferation and DNA replication. In fact, certain oncogenes are able to induce replication stress and promote genomic instability in human carcinogenesis (Primo and Teixeira, 2019). One such example is oncogenic Cyclin E/CDK2 complex. Hyperactivation of Cyclin E/CDK2 causes genomic instability in different experimental models and correlates with an increased frequency of genomic alterations in human cancers (Teixeira and Reed, 2017). Furthermore, high levels of Cyclin E and/or increased activity of CDK2 have been observed in numerous cancers, ranging from hematological malignancies to numerous solid tumors, and are associated with poor prognosis and decreased survival in cancer patients (Hwang and Clurman, 2005).

High levels of Cyclin E1 protein have been initially shown to induce chromosome gains and losses, causing aneuploidy in normal human mammary epithelial cells (Spruck et al., 1999). Consistently, either *FBXW7* deletion or *SKP1* silencing, which results in accumulation of Cyclin E protein among other oncoproteins, led to increased micronucleus formation, multipolar spindles, and chromosome instability in human colorectal cancer cells (Rajagopalan et al., 2004; Thompson et al., 2020). These findings were associated with increased levels of Cyclin E1 protein, as additional *CCNE1* silencing in *FBXW7-* or *SKP1*-deficient cells partially rescued micronucleus formation. Importantly, the effects of high levels of Cyclin E1 on chromosome instability seem to depend on CDK2 activity, as a hyperactive *CDK2* knockin allele was sufficient to induce increased Cyclin E1-associated CDK2 activity and micronucleus formation in human colorectal cancer cells (Hughes et al., 2013). *CCNE1* and *CCNE2* amplification, as well as increased mRNA expression, have been also correlated with whole genome doublings in human cancers (Zack et al., 2013; Lee et al., 2020). One of the proposed mechanisms to explain how hyperactive Cyclin E/CDK2 causes genome doublings is the induction of mitotic failure or endoreduplication, with subsequent formation of polyploid cells. Indeed, high levels of Cyclin E1 have been shown to impair mitotic progression and cause accumulation of cells in early stages of mitosis. This is caused by Cyclin E1/CDK2-mediated phosphorylation and inactivation of the APC/C adaptor protein CDH1, followed by accumulation of Cyclin B1 and Securin, and eventually mitotic failure (Keck et al., 2007). In agreement with these findings, precise regulation of Cyclin E protein is critical to normal endocycles, as *CCNE1/2*-double deficient mice present defects in endoreduplication of megakaryocytes and placental trophoblast giant cells (Geng et al., 2003; Parisi et al., 2003).

Another potential mechanism to explain how oncogenic Cyclin E/CDK2 interferes with normal ploidy and causes genomic instability is centrosome amplification, which induces the formation of merotelic kinetochore-microtubule attachments and ultimately leads to aberrant chromosome segregation (Godinho and Pellman, 2014). As mentioned before, Cyclin E1 localizes to centrosomes and, together with CDK2, is able to phosphorylate several centrosome proteins, such as NPM, CP110, and MPS1, regulating the process of centrosome duplication (Okuda et al., 2000; Fisk and Winey, 2001; Tokuyama et al., 2001; Chen et al., 2002; Matsumoto and Maller, 2004). Even though high levels of Cyclin E1 alone are not sufficient to induce centrosome amplification in human cells (Spruck et al., 1999), it does synergize with *TP53* loss to cause centrosome amplification and chromosome segregation errors (Mussman et al., 2000; Kawamura et al., 2004). Accordingly, a hyperactive *CDK2* knockin allele mouse model was sufficient to induce increased centrosome numbers (Zhao et al., 2012). It is possible that Cyclin E/CDK2 hyperactivation impairs the localization and function of certain centrosome proteins, causing centrosome amplification and subsequent chromosomal gains and/or losses. In fact, oncogenic Cyclin E1/CDK2 aberrantly hyperphosphorylates centromere protein A (CENPA), reducing CENPA localization at centromeres and causing chromosome missegregation and increased micronucleus formation (Takada et al., 2017).

Apart from whole chromosome gains/losses, oncogenic activation of Cyclin E/CDK2 has been shown to cause copy number alterations at specific genomic segments (Costantino et al., 2014; Miron et al., 2015; Teixeira et al., 2015; Menghi et al., 2018; Giraldez et al., 2019; Kok et al., 2020). It has been demonstrated that Cyclin E1/CDK2 hyperactivation impairs S phase progression, allowing cells to enter into mitosis with unreplicated genomic regions. Incompletely replicated chromosomal segments, in turn, lead to segregation abnormalities in mitosis and eventually genomic deletions. In fact, *CCNE1* amplification associates with copy number losses at specific genomic sites in human breast cancers (Teixeira et al., 2015). It has been shown that large genomic deletions may contribute to oncogenesis by promoting loss of heterozygosity at tumor suppressor genes and deletion of fragile sites (Bignell et al., 2010). More complex mechanisms have been also shown to contribute to Cyclin E/CDK2-induced genomic instability. It has been indicated that Cyclin E1-induced replication fork collapse during S phase can be repaired by break-induced replication (BIR), generating segmental tandem duplications as a consequence of BIR (Costantino et al., 2014). Again, *CCNE1* amplification has been associated with tandem duplications in different human cancers, mostly favoring oncogenesis by causing oncogene duplication (Menghi et al., 2018). Besides copy number alterations, *CCNE1* amplification has been also associated with chromosomal breakpoints and rearrangements in human cancers (Macheret and Halazonetis, 2018). Together, these data indicate that oncogenic activation of Cyclin E/CDK2 complex drives numerous forms of genomic instability in human cancers, ranging from whole chromosomal gains and/or losses to focal genomic deletions and/or amplifications, as well as chromosome rearrangements.

## Concluding Remarks and Future Perspectives

Uncontrolled cell proliferation and abnormal activity of cell cycle proteins are at the basis of carcinogenesis. As a result, various cell cycle regulators have been considered as potential targets in cancer therapy (Otto and Sicinski, 2017; Suski et al., 2021). Normal activity of Cyclin E/CDK2 complex is essential for appropriate cell cycle progression and DNA replication. On the other hand, oncogenic activation of Cyclin E/CDK2 complex has been shown to interfere with DNA replication and cause replication stress through several different mechanisms. Hyperactivation of Cyclin E/CDK2 induces genomic instability in human cancers, typified by the increased frequency of chromosomal gains and/or losses and rearrangements. High levels of Cyclin E and/or increased CDK2 activity are associated with poor clinical outcome and decreased survival in cancer patients. Therefore, targeting oncogenic activity of Cyclin E/CDK2 complex (e.g. through Cyclin E-targeted degradation or CDK2-selective inhibition) may represent an attractive therapeutic strategy for specific cancer subtypes, such as breast, uterine, and ovarian cancers (Tadesse et al., 2020; Suski et al., 2021).

Oncogene activation causes numerous alterations in tumor cells. Along the tumorigenic process, oncogenic insults may uncover potential cancer vulnerabilities. In fact, oncogene-induced replication stress leads to activation of the RSR pathway, which has been proposed to represent a promising target in cancer therapy (Ngoi et al., 2021). Due to the excessive level of replication stress caused by activation of certain oncogenes, cancer cells heavily rely upon RSR pathway activation in order to survive. Indeed, several screening approaches have identified synthetic lethal interactions between oncogene activation and inhibition of replication checkpoint proteins. Cancer cells experiencing high levels of Cyclin E protein and/or high CDK2 activity have shown increased sensitivity to inhibitors of RSR protein kinases, such as ATR, CHK1, and WEE1 (Murga et al., 2011; Toledo et al., 2011; Chen et al., 2018; Kok et al., 2020; Sviderskiy et al., 2020). Combination of Cyclin E/CDK2 hyperactivation and inhibition of such protein kinases leads to irreversible DNA damage and selective death of cancer cells in different models. More recently, *CCNE1* amplification has been also identified to be synthetic lethal with inhibition of PKMYT1 kinase, a negative regulator of CDK1 (Gallo et al., 2021). The results suggest that further activation of CDK1 is not compatible with oncogenic Cyclin E/CDK2 environments, leading to unscheduled mitotic entry and genome instability. Altogether, these data indicate that cancer subtypes with oncogenic activation of Cyclin E/CDK2 complex and subsequent robust activation of RSR may be especially vulnerable and uniquely sensitive to inhibitors of replication checkpoint proteins. Exploiting these therapeutic opportunities will certainly prove beneficial to cancer treatment in the following years.

## References

[B1] Adams P. D. Sellers W. R. Sharma S. K. Wu A. D. Nalin C. M. Kaelin W. G. Jr (1996). Identification of a Cyclin-Cdk2 Recognition Motif Present in Substrates and P21-like Cyclin-dependent Kinase Inhibitors. Mol. Cell. Biol. 16, 6623–6633. 10.1128/MCB.16.12.6623 8943316 PMC231664

[B2] Aguilera A. García-Muse T. (2013). Causes of Genome Instability. Annu. Rev. Genet. 47, 1–32. 10.1146/annurev-genet-111212-133232 23909437

[B3] Ait-Si-Ali S. Ramirez S. Barre F.-X. Dkhissi F. Magnaghi-Jaulin L. Girault J. A. (1998). Histone Acetyltransferase Activity of CBP Is Controlled by Cycle-dependent Kinases and Oncoprotein E1A. Nature 396, 184–186. 10.1038/24190 9823900

[B4] Aleem E. Kiyokawa H. Kaldis P. (2005). Cdc2-cyclin E Complexes Regulate the G1/S Phase Transition. Nat. Cell Biol. 7, 831–836. 10.1038/ncb1284 16007079

[B5] Anglana M. Apiou F. Bensimon A. Debatisse M. (2003). Dynamics of DNA Replication in Mammalian Somatic Cells. Cell 114, 385–394. 10.1016/s0092-8674(03)00569-5 12914702

[B6] Bartkova J. Hořejší Z. Koed K. Krämer A. Tort F. Zieger K. (2005). DNA Damage Response as a Candidate Anti-cancer Barrier in Early Human Tumorigenesis. Nature 434, 864–870. 10.1038/nature03482 15829956

[B7] Bartkova J. Rezaei N. Liontos M. Karakaidos P. Kletsas D. Issaeva N. (2006). Oncogene-induced Senescence Is Part of the Tumorigenesis Barrier Imposed by DNA Damage Checkpoints. Nature 444, 633–637. 10.1038/nature05268 17136093

[B8] Bellelli R. Boulton S. J. (2021). Spotlight on the Replisome: Aetiology of DNA Replication-Associated Genetic Diseases. Trends Genet. 37, 317–336. 10.1016/j.tig.2020.09.008 33041047

[B9] Bester A. C. Roniger M. Oren Y. S. Im M. M. Sarni D. Chaoat M. (2011). Nucleotide Deficiency Promotes Genomic Instability in Early Stages of Cancer Development. Cell 145, 435–446. 10.1016/j.cell.2011.03.044 21529715 PMC3740329

[B10] Bignell G. R. Greenman C. D. Davies H. Butler A. P. Edkins S. Andrews J. M. (2010). Signatures of Mutation and Selection in the Cancer Genome. Nature 463, 893–898. 10.1038/nature08768 20164919 PMC3145113

[B11] Boos D. Sanchez-Pulido L. Rappas M. Pearl L. H. Oliver A. W. Ponting C. P. (2011). Regulation of DNA Replication through Sld3-Dpb11 Interaction Is Conserved from Yeast to Humans. Curr. Biol. 21, 1152–1157. 10.1016/j.cub.2011.05.057 21700459

[B12] Burgers P. M. J. Kunkel T. A. (2017). Eukaryotic DNA Replication fork. Annu. Rev. Biochem. 86, 417–438. 10.1146/annurev-biochem-061516-044709 28301743 PMC5597965

[B13] Caldon C. Musgrove E. A. (2010). Distinct and Redundant Functions of Cyclin E1 and Cyclin E2 in Development and Cancer. Cell Div. 5, 2. 10.1186/1747-1028-5-2 20180967 PMC2835679

[B14] Caruso J. A. Duong M. T. Carey J. P. W. Hunt K. K. Keyomarsi K. (2018). Low-molecular-weight Cyclin E in Human Cancer: Cellular Consequences and Opportunities for Targeted Therapies. Cancer Res. 78, 5481–5491. 10.1158/0008-5472.CAN-18-1235 30194068 PMC6168358

[B15] Chen J. Saha P. Kornbluth S. Dynlacht B. D. Dutta A. (1996). Cyclin-binding Motifs Are Essential for the Function of p21CIP1. Mol. Cell. Biol. 16, 4673–4682. 10.1128/MCB.16.9.4673 8756624 PMC231467

[B16] Chen X. Low K.-H. Alexander A. Jiang Y. Karakas C. Hess K. R. (2018). Cyclin E Overexpression Sensitizes Triple-Negative Breast Cancer to Wee1 Kinase Inhibition. Clin. Cancer Res. 24, 6594–6610. 10.1158/1078-0432.CCR-18-1446 30181387 PMC6317865

[B17] Chen Z. Indjeian V. B. McManus M. Wang L. Dynlacht B. D. (2002). CP110, a Cell Cycle-dependent CDK Substrate, Regulates Centrosome Duplication in Human Cells. Dev. Cell 3, 339–350. 10.1016/s1534-5807(02)00258-7 12361598

[B18] Chi Y. Carter J. H. Swanger J. Mazin A. V. Moritz R. L. Clurman B. E. (2020). A Novel Landscape of Nuclear Human CDK2 Substrates Revealed by *In Situ* Phosphorylation. Sci. Adv. 6, eaaz9899. 10.1126/sciadv.aaz9899 32494624 PMC7164936

[B19] Chu C. Geng Y. Zhou Y. Sicinski P. (2021). Cyclin E in normal Physiology and Disease States. Trends Cell Biol. 31, 732–746. 10.1016/j.tcb.2021.05.001 34052101 PMC8364501

[B20] Chuang L.-C. Teixeira L. K. Wohlschlegel J. A. Henze M. Yates J. R. Méndez J. (2009). Phosphorylation of Mcm2 by Cdc7 Promotes Pre-replication Complex Assembly during Cell-Cycle Re-entry. Mol. Cell 35, 206–216. 10.1016/j.molcel.2009.06.014 19647517 PMC2725784

[B21] Clurman B. E. Sheaff R. J. Thress K. Groudine M. Roberts J. M. (1996). Turnover of Cyclin E by the Ubiquitin-Proteasome Pathway Is Regulated by Cdk2 Binding and Cyclin Phosphorylation. Genes Dev. 10, 1979–1990. 10.1101/gad.10.16.1979 8769642

[B22] Cook J. G. Park C.-H. Burke T. W. Leone G. DeGregori J. Engel A. (2002). Analysis of Cdc6 Function in the Assembly of Mammalian Prereplication Complexes. Proc. Natl. Acad. Sci. 99, 1347–1352. 10.1073/pnas.032677499 11805305 PMC122193

[B23] Costantino L. Sotiriou S. K. Rantala J. K. Magin S. Mladenov E. Helleday T. (2014). Break-induced Replication Repair of Damaged forks Induces Genomic Duplications in Human Cells. Science 343, 88–91. 10.1126/science.1243211 24310611 PMC4047655

[B24] Coverley D. Laman H. Laskey R. A. (2002). Distinct Roles for Cyclins E and A during DNA Replication Complex Assembly and Activation. Nat. Cell Biol. 4, 523–528. 10.1038/ncb813 12080347

[B25] Dowdy S. F. Hinds P. W. Louie K. Reed S. I. Arnold A. Weinberg R. A. (1993). Physical Interaction of the Retinoblastoma Protein with Human D Cyclins. Cell 73, 499–511. 10.1016/0092-8674(93)90137-f 8490963

[B26] Dulic V. Lees E. Reed S. (1992). Association of Human Cyclin E with a Periodic G1-S Phase Protein Kinase. Science 257, 1958–1961. 10.1126/science.1329201 1329201

[B27] Ekholm-Reed S. Méndez J. Tedesco D. Zetterberg A. Stillman B. Reed S. I. (2004). Deregulation of Cyclin E in Human Cells Interferes with Prereplication Complex Assembly. J. Cell Biol. 165, 789–800. 10.1083/jcb.200404092 15197178 PMC2172392

[B28] Fisk H. A. Winey M. (2001). The Mouse Mps1p-like Kinase Regulates Centrosome Duplication. Cell 106, 95–104. 10.1016/s0092-8674(01)00411-1 11461705

[B29] Fragkos M. Ganier O. Coulombe P. Méchali M. (2015). DNA Replication Origin Activation in Space and Time. Nat. Rev. Mol. Cell Biol. 16, 360–374. 10.1038/nrm4002 25999062

[B30] Furstenthal L. Kaiser B. K. Swanson C. Jackson P. K. (2001). Cyclin E Uses Cdc6 as a Chromatin-Associated Receptor Required for DNA Replication. J. Cell Biol. 152, 1267–1278. 10.1083/jcb.152.6.1267 11257126 PMC2199215

[B31] Gaillard H. García-Muse T. Aguilera A. (2015). Replication Stress and Cancer. Nat. Rev. Cancer 15, 276–289. 10.1038/nrc3916 25907220

[B32] Gallo D. Young J. T. F. Fourtounis J. Martino G. Álvarez-Quilón A. Bernier C. (2021). CCNE1 Amplification Is Synthetic-Lethal with PKMYT1 Kinase Inhibition. *bioRxiv* [Preprint]. 10.1101/2021.04.08.438361 PMC9046089 35444283

[B33] Geng Y. Eaton E. N. Picón M. Roberts J. M. Lundberg A. S. Gifford A. (1996). Regulation of Cyclin E Transcription by E2Fs and Retinoblastoma Protein. Oncogene 12, 1173–1180. 8649818

[B34] Geng Y. Lee Y.-M. Welcker M. Swanger J. Zagozdzon A. Winer J. D. (2007). Kinase-independent Function of Cyclin E. Mol. Cell 25, 127–139. 10.1016/j.molcel.2006.11.029 17218276

[B35] Geng Y. Yu Q. Sicinska E. Das M. Schneider J. E. Bhattacharya S. (2003). Cyclin E Ablation in the Mouse. Cell 114, 431–443. 10.1016/s0092-8674(03)00645-7 12941272

[B36] Giangrande P. H. Zhu W. Schlisio S. Sun X. Mori S. Gaubatz S. (2004). A Role for E2F6 in Distinguishing G1/S- and G2/M-specific Transcription. Genes Dev. 18, 2941–2951. 10.1101/gad.1239304 15574595 PMC534654

[B37] Giraldez S. Tamayo P. Wineinger N. Kim W. Reed S. I. (2019). Cyclin E Overexpression in Human Mammary Epithelial Cells Promotes Epithelial Cancer-specific Copy Number Alterations. iScience 19, 850–859. 10.1016/j.isci.2019.08.043 31513970 PMC6739637

[B38] Godinho S. A. Pellman D. (2014). Causes and Consequences of Centrosome Abnormalities in Cancer. Phil. Trans. R. Soc. B 369, 20130467. 10.1098/rstb.2013.0467 25047621 PMC4113111

[B75] Gudas J. M. Payton M. Thukral S. Chen E. Bass M. Robinson M. O. (1999). Cyclin E2, a Novel G 1 Cyclin that Binds Cdk2 and Is Aberrantly Expressed in Human Cancers. Mol. Cell. Biol. 19, 612–622. 10.1128/MCB.19.1.612 9858585 PMC83919

[B39] Hall C. Nelson D. M. Ye X. Baker K. DeCaprio J. A. Seeholzer S. (2001). HIRA, the Human Homologue of Yeast Hir1p and Hir2p, Is a Novel Cyclin-Cdk2 Substrate Whose Expression Blocks S-phase Progression. Mol. Cell. Biol. 21, 1854–1865. 10.1128/MCB.21.5.1854-1865.2001 11238922 PMC86753

[B40] Harbour J. W. Dean D. C. (2000). The Rb/E2F Pathway: Expanding Roles and Emerging Paradigms. Genes Dev. 14, 2393–2409. 10.1101/gad.813200 11018009

[B41] Helmrich A. Ballarino M. Nudler E. Tora L. (2013). Transcription-replication Encounters, Consequences and Genomic Instability. Nat. Struct. Mol. Biol. 20, 412–418. 10.1038/nsmb.2543 23552296

[B42] Honda R. Lowe E. D. Dubinina E. Skamnaki V. Cook A. Brown N. R. (2005). The Structure of Cyclin E1/CDK2: Implications for CDK2 Activation and CDK2-independent Roles. EMBO J. 24, 452–463. 10.1038/sj.emboj.7600554 15660127 PMC548659

[B43] Hossain M. Bhalla K. Stillman B. (2021). Multiple, Short Protein Binding Motifs in ORC1 and CDC6 Control the Initiation of DNA Replication. Mol. Cell 81, 1951–1969. 10.1016/j.molcel.2021.03.003 33761311 PMC8106667

[B44] Hossain M. Stillman B. (2016). Opposing Roles for DNA Replication Initiator Proteins ORC1 and CDC6 in Control of Cyclin E Gene Transcription. Elife 5, e12785. 10.7554/eLife.12785 27458800 PMC4987141

[B45] Hsu J. Y. Reimann J. D. R. Sørensen C. S. Lukas J. Jackson P. K. (2002). E2F-dependent Accumulation of hEmi1 Regulates S Phase Entry by Inhibiting APCCdh1. Nat. Cell Biol. 4, 358–366. 10.1038/ncb785 11988738

[B46] Hughes B. T. Sidorova J. Swanger J. Monnat R. J. Jr Clurman B. E. (2013). Essential Role for Cdk2 Inhibitory Phosphorylation during Replication Stress Revealed by a Human Cdk2 Knockin Mutation. Proc. Natl. Acad. Sci. 110, 8954–8959. 10.1073/pnas.1302927110 23671119 PMC3670391

[B47] Hwang H. C. Clurman B. E. (2005). Cyclin E in normal and Neoplastic Cell Cycles. Oncogene 24, 2776–2786. 10.1038/sj.onc.1208613 15838514

[B48] Ishida S. Huang E. Zuzan H. Spang R. Leone G. West M. (2001). Role for E2F in Control of Both DNA Replication and Mitotic Functions as Revealed from DNA Microarray Analysis. Mol. Cell. Biol. 21, 4684–4699. 10.1128/MCB.21.14.4684-4699.2001 11416145 PMC87143

[B49] Jackman M. Kubota Y. den Elzen N. Hagting A. Pines J. (2002). Cyclin A- and Cyclin E-Cdk Complexes Shuttle between the Nucleus and the Cytoplasm. Mol. Biol. Cell 13, 1030–1045. 10.1091/mbc.01-07-0361 11907280 PMC99617

[B50] Jones R. M. Mortusewicz O. Afzal I. Lorvellec M. García P. Helleday T. (2013). Increased Replication Initiation and Conflicts with Transcription Underlie Cyclin E-Induced Replication Stress. Oncogene 32, 3744–3753. 10.1038/onc.2012.387 22945645

[B51] Kawamura K. Izumi H. Ma Z. Ikeda R. Moriyama M. Tanaka T. (2004). Induction of Centrosome Amplification and Chromosome Instability in Human Bladder Cancer Cells by P53 Mutation and Cyclin E Overexpression. Cancer Res. 64, 4800–4809. 10.1158/0008-5472.CAN-03-3908 15256449

[B52] Keck J. M. Summers M. K. Tedesco D. Ekholm-Reed S. Chuang L.-C. Jackson P. K. (2007). Cyclin E Overexpression Impairs Progression through Mitosis by Inhibiting APCCdh1. J. Cell Biol. 178, 371–385. 10.1083/jcb.200703202 17664332 PMC2064850

[B53] Kelly B. L. Wolfe K. G. Roberts J. M. (1998). Identification of a Substrate-Targeting Domain in Cyclin E Necessary for Phosphorylation of the Retinoblastoma Protein. Proc. Natl. Acad. Sci. 95, 2535–2540. 10.1073/pnas.95.5.2535 9482921 PMC19404

[B54] Koepp D. M. Schaefer L. K. Ye X. Keyomarsi K. Chu C. Harper J. W. (2001). Phosphorylation-Dependent Ubiquitination of Cyclin E by the SCF Fbw7 Ubiquitin Ligase. Science 294, 173–177. 10.1126/science.1065203 11533444

[B55] Koff A. Cross F. Fisher A. Schumacher J. Leguellec K. Philippe M. (1991). Human Cyclin E, a New Cyclin that Interacts with Two Members of the CDC2 Gene Family. Cell 66, 1217–1228. 10.1016/0092-8674(91)90044-y 1833068

[B56] Koff A. Giordano A. Desai D. Yamashita K. Harper J. W. Elledge S. (1992). Formation and Activation of a Cyclin E-Cdk2 Complex during the G 1 Phase of the Human Cell Cycle. Science 257, 1689–1694. 10.1126/science.1388288 1388288

[B57] Kok Y. P. Guerrero Llobet S. Schoonen P. M. Everts M. Bhattacharya A. Fehrmann R. S. N. (2020). Overexpression of Cyclin E1 or Cdc25A Leads to Replication Stress, Mitotic Aberrancies, and Increased Sensitivity to Replication Checkpoint Inhibitors. Oncogenesis 9, 88. 10.1038/s41389-020-00270-2 33028815 PMC7542455

[B58] Kumagai A. Shevchenko A. Shevchenko A. Dunphy W. G. (2011). Direct Regulation of Treslin by Cyclin-dependent Kinase Is Essential for the Onset of DNA Replication. J. Cell Biol. 193, 995–1007. 10.1083/jcb.201102003 21646402 PMC3115804

[B59] Kumagai A. Shevchenko A. Shevchenko A. Dunphy W. G. (2010). Treslin Collaborates with TopBP1 in Triggering the Initiation of DNA Replication. Cell 140, 349–359. 10.1016/j.cell.2009.12.049 20116089 PMC2857569

[B60] Lane A. N. Fan T. W.-M. (2015). Regulation of Mammalian Nucleotide Metabolism and Biosynthesis. Nucleic Acids Res. 43, 2466–2485. 10.1093/nar/gkv047 25628363 PMC4344498

[B61] Lauper N. Beck A. R. Cariou S. Richman L. Hofmann K. Reith W. (1998). Cyclin E2: a Novel CDK2 Partner in the Late G1 and S Phases of the Mammalian Cell Cycle. Oncogene 17, 2637–2643. 10.1038/sj.onc.1202477 9840927

[B62] Lee C. Fernandez K. J. Alexandrou S. Sergio C. M. Deng N. Rogers S. (2020). Cyclin E2 Promotes Whole Genome Doubling in Breast Cancer. Cancers 12, 2268. 10.3390/cancers12082268 PMC7463708 32823571

[B63] Lew D. J. Dulić V. Reed S. I. (1991). Isolation of Three Novel Human Cyclins by rescue of G1 Cyclin (Cln) Function in Yeast. Cell 66, 1197–1206. 10.1016/0092-8674(91)90042-w 1833066

[B64] Liberal V. Martinsson-Ahlzén H.-S. Liberal J. Spruck C. H. Widschwendter M. McGowan C. H. (2012). Cyclin-dependent Kinase Subunit (Cks) 1 or Cks2 Overexpression Overrides the DNA Damage Response Barrier Triggered by Activated Oncoproteins. Proc. Natl. Acad. Sci. 109, 2754–2759. 10.1073/pnas.1102434108 21697511 PMC3286935

[B65] Lu H. Shamanna R. A. de Freitas J. K. Okur M. Khadka P. Kulikowicz T. (2017). Cell Cycle-dependent Phosphorylation Regulates RECQL4 Pathway Choice and Ubiquitination in DNA Double-Strand Break Repair. Nat. Commun. 8, 2039. 10.1038/s41467-017-02146-3 29229926 PMC5725494

[B66] Ma T. Van Tine B. A. Wei Y. Garrett M. D. Nelson D. Adams P. D. (2000). Cell Cycle-Regulated Phosphorylation of p220NPAT by Cyclin E/Cdk2 in Cajal Bodies Promotes Histone Gene Transcription. Genes Dev. 14, 2298–2313. 10.1101/gad.829500 10995387 PMC316935

[B67] Macheret M. Halazonetis T. D. (2018). Intragenic Origins Due to Short G1 Phases Underlie Oncogene-Induced DNA Replication Stress. Nature 555, 112–116. 10.1038/nature25507 29466339 PMC5837010

[B68] Mailand N. Diffley J. F. X. (2005). CDKs Promote DNA Replication Origin Licensing in Human Cells by Protecting Cdc6 from APC/C-dependent Proteolysis. Cell 122, 915–926. 10.1016/j.cell.2005.08.013 16153703

[B69] Malumbres M. Barbacid M. (2009). Cell Cycle, CDKs and Cancer: a Changing Paradigm. Nat. Rev. Cancer 9, 153–166. 10.1038/nrc2602 19238148

[B70] Masai H. Matsumoto S. You Z. Yoshizawa-Sugata N. Oda M. (2010). Eukaryotic Chromosome DNA Replication: where, when, and How? Annu. Rev. Biochem. 79, 89–130. 10.1146/annurev.biochem.052308.103205 20373915

[B71] Matsumoto Y. Maller J. L. (2004). A Centrosomal Localization Signal in Cyclin E Required for Cdk2-independent S Phase Entry. Science 306, 885–888. 10.1126/science.1103544 15514162

[B72] Matthews H. K. Bertoli C. de Bruin R. A. M. (2021). Cell Cycle Control in Cancer. Nat. Rev. Mol. Cell Biol. 10.1038/s41580-021-00404-3 34508254

[B73] McIntosh D. Blow J. J. (2012). Dormant Origins, the Licensing Checkpoint, and the Response to Replicative Stresses. Cold Spring Harbor Perspect. Biol. 4, a012955. 10.1101/cshperspect.a012955 PMC3475168 22904560

[B74] Menghi F. Barthel F. P. Yadav V. Tang M. Ji B. Tang Z. (2018). The Tandem Duplicator Phenotype Is a Prevalent Genome-wide Cancer Configuration Driven by Distinct Gene Mutations. Cancer Cell 34, 197–210. 10.1016/j.ccell.2018.06.008 30017478 PMC6481635

[B76] Miron K. Golan-Lev T. Dvir R. Ben-David E. Kerem B. (2015). Oncogenes Create a Unique Landscape of Fragile Sites. Nat. Commun. 6, 7094. 10.1038/ncomms8094 25959793

[B77] Moore J. D. Kornbluth S. Hunt T. (2002). Identification of the Nuclear Localization Signal inXenopusCyclin E and Analysis of its Role in Replication and Mitosis. Mol. Biol. Cell 13, 4388–4400. 10.1091/mbc.e02-07-0449 12475960 PMC138641

[B78] Morris L. Allen K. E. La Thangue N. B. (2000). Regulation of E2F Transcription by Cyclin E-Cdk2 Kinase Mediated through P300/CBP Co-activators. Nat. Cell Biol. 2, 232–239. 10.1038/35008660 10783242

[B79] Murga M. Campaner S. Lopez-Contreras A. J. Toledo L. I. Soria R. Montaña M. F. (2011). Exploiting Oncogene-Induced Replicative Stress for the Selective Killing of Myc-Driven Tumors. Nat. Struct. Mol. Biol. 18, 1331–1335. 10.1038/nsmb.2189 22120667 PMC4894468

[B80] Mussman J. G. Horn H. F. Carroll P. E. Okuda M. Tarapore P. Donehower L. A. (2000). Synergistic Induction of Centrosome Hyperamplification by Loss of P53 and Cyclin E Overexpression. Oncogene 19, 1635–1646. 10.1038/sj.onc.1203460 10763820

[B81] Neelsen K. J. Zanini I. M. Y. Herrador R. Lopes M. (2013). Oncogenes Induce Genotoxic Stress by Mitotic Processing of Unusual Replication Intermediates. J. Cell Biol. 200, 699–708. 10.1083/jcb.201212058 23479741 PMC3601361

[B82] Negrini S. Gorgoulis V. G. Halazonetis T. D. (2010). Genomic Instability - an Evolving Hallmark of Cancer. Nat. Rev. Mol. Cell Biol. 11, 220–228. 10.1038/nrm2858 20177397

[B83] Ngoi N. Y. L. Pham M. M. Tan D. S. P. Yap T. A. (2021). Targeting the Replication Stress Response through Synthetic Lethal Strategies in Cancer Medicine. Trends Cancer 7, 930–957. 10.1016/j.trecan.2021.06.002 34215565 PMC8458263

[B84] Odajima J. Saini S. Jung P. Ndassa-Colday Y. Ficaro S. Geng Y. (2016). Proteomic Landscape of Tissue-specific Cyclin E Functions *In Vivo* . Plos Genet. 12, e1006429. 10.1371/journal.pgen.1006429 27828963 PMC5102403

[B85] Ohtani K. DeGregori J. Nevins J. R. (1995). Regulation of the Cyclin E Gene by Transcription Factor E2F1. Proc. Natl. Acad. Sci. 92, 12146–12150. 10.1073/pnas.92.26.12146 8618861 PMC40313

[B86] Ohtsubo M. Roberts J. M. (1993). Cyclin-Dependent Regulation of G 1 in Mammalian Fibroblasts. Science 259, 1908–1912. 10.1126/science.8384376 8384376

[B87] Okuda M. Horn H. F. Tarapore P. Tokuyama Y. Smulian A. G. Chan P.-K. (2000). Nucleophosmin/B23 Is a Target of CDK2/cyclin E in Centrosome Duplication. Cell 103, 127–140. 10.1016/s0092-8674(00)00093-3 11051553

[B88] Otto T. Sicinski P. (2017). Cell Cycle Proteins as Promising Targets in Cancer Therapy. Nat. Rev. Cancer 17, 93–115. 10.1038/nrc.2016.138 28127048 PMC5345933

[B89] Pagliuca F. W. Collins M. O. Lichawska A. Zegerman P. Choudhary J. S. Pines J. (2011). Quantitative Proteomics Reveals the Basis for the Biochemical Specificity of the Cell-Cycle Machinery. Mol. Cell 43, 406–417. 10.1016/j.molcel.2011.05.031 21816347 PMC3332305

[B90] Parisi T. Beck A. R. Rougier N. McNeil T. Lucian L. Werb Z. (2003). Cyclins E1 and E2 Are Required for Endoreplication in Placental Trophoblast Giant Cells. EMBO J. 22, 4794–4803. 10.1093/emboj/cdg482 12970191 PMC212738

[B91] Polager S. Kalma Y. Berkovich E. Ginsberg D. (2002). E2Fs Up-Regulate Expression of Genes Involved in DNA Replication, DNA Repair and Mitosis. Oncogene 21, 437–446. 10.1038/sj.onc.1205102 11821956

[B92] Primo L. M. F. Teixeira L. K. (2019). DNA Replication Stress: Oncogenes in the Spotlight. Genet. Mol. Biol. 43, e20190138. 10.1590/1678-4685GMB-2019-0138 31930281 PMC7197996

[B93] Rajagopalan H. Jallepalli P. V. Rago C. Velculescu V. E. Kinzler K. W. Vogelstein B. (2004). Inactivation of hCDC4 Can Cause Chromosomal Instability. Nature 428, 77–81. 10.1038/nature02313 14999283

[B94] Resnitzky D. Gossen M. Bujard H. Reed S. I. (1994). Acceleration of the G1/S Phase Transition by Expression of Cyclins D1 and E with an Inducible System. Mol. Cell. Biol. 14, 1669–1679. 10.1128/mcb.14.3.1669-1679.1994 8114703 PMC358525

[B95] Sansam C. G. Goins D. Siefert J. C. Clowdus E. A. Sansam C. L. (2015). Cyclin-dependent Kinase Regulates the Length of S Phase through TICRR/TRESLIN Phosphorylation. Genes Dev. 29, 555–566. 10.1101/gad.246827.114 25737283 PMC4358407

[B96] Schulman B. A. Lindstrom D. L. Harlow E. (1998). Substrate Recruitment to Cyclin-dependent Kinase 2 by a Multipurpose Docking Site on Cyclin A. Proc. Natl. Acad. Sci. 95, 10453–10458. 10.1073/pnas.95.18.10453 9724724 PMC27915

[B97] Sheaff R. J. Groudine M. Gordon M. Roberts J. M. Clurman B. E. (1997). Cyclin E-CDK2 Is a Regulator of p27Kip1. Genes Dev. 11, 1464–1478. 10.1101/gad.11.11.1464 9192873

[B98] Sherr C. J. Roberts J. M. (2004). Living with or without Cyclins and Cyclin-dependent Kinases. Genes Dev. 18, 2699–2711. 10.1101/gad.1256504 15545627

[B99] Siddiqui K. On K. F. Diffley J. F. X. (2013). Regulating DNA Replication in Eukarya. Cold Spring Harbor Perspect. Biol. 5, a012930. 10.1101/cshperspect.a012930 PMC3753713 23838438

[B100] Siu K. T. Rosner M. R. Minella A. C. (2012). An Integrated View of Cyclin E Function and Regulation. Cell Cycle 11, 57–64. 10.4161/cc.11.1.18775 22186781 PMC3272232

[B101] Spruck C. H. Won K.-A. Reed S. I. (1999). Deregulated Cyclin E Induces Chromosome Instability. Nature 401, 297–300. 10.1038/45836 10499591

[B102] Strohmaier H. Spruck C. H. Kaiser P. Won K.-A. Sangfelt O. Reed S. I. (2001). Human F-Box Protein hCdc4 Targets Cyclin E for Proteolysis and Is Mutated in a Breast Cancer Cell Line. Nature 413, 316–322. 10.1038/35095076 11565034

[B103] Suski J. M. Braun M. Strmiska V. Sicinski P. (2021). Targeting Cell-Cycle Machinery in Cancer. Cancer Cell 39, 759–778. 10.1016/j.ccell.2021.03.010 33891890 PMC8206013

[B104] Sviderskiy V. O. Blumenberg L. Gorodetsky E. Karakousi T. R. Hirsh N. Alvarez S. W. (2020). Hyperactive CDK2 Activity in Basal-like Breast Cancer Imposes a Genome Integrity Liability that Can Be Exploited by Targeting DNA Polymerase ε. Mol. Cell 80, 682–698. 10.1016/j.molcel.2020.10.016 33152268 PMC7687292

[B105] Tadesse S. Anshabo A. T. Portman N. Lim E. Tilley W. Caldon C. E. (2020). Targeting CDK2 in Cancer: Challenges and Opportunities for Therapy. Drug Discov. Today 25, 406–413. 10.1016/j.drudis.2019.12.001 31839441

[B106] Takada M. Zhang W. Suzuki A. Kuroda T. S. Yu Z. Inuzuka H. (2017). FBW7 Loss Promotes Chromosomal Instability and Tumorigenesis via Cyclin E1/CDK2-Mediated Phosphorylation of CENP-A. Cancer Res. 77, 4881–4893. 10.1158/0008-5472.CAN-17-1240 28760857 PMC5743019

[B107] Tanaka S. Araki H. (2013). Helicase Activation and Establishment of Replication forks at Chromosomal Origins of Replication. Cold Spring Harbor Perspect. Biol. 5, a010371. 10.1101/cshperspect.a010371 PMC3839609 23881938

[B108] Tanaka S. Diffley J. F. (2002). Deregulated G1-Cyclin Expression Induces Genomic Instability by Preventing Efficient Pre-RC Formation. Genes Dev. 16, 2639–2649. 10.1101/gad.1011002 12381663 PMC187461

[B109] Tanaka S. Umemori T. Hirai K. Muramatsu S. Kamimura Y. Araki H. (2007). CDK-dependent Phosphorylation of Sld2 and Sld3 Initiates DNA Replication in Budding Yeast. Nature 445, 328–332. 10.1038/nature05465 17167415

[B110] Teixeira L. K. Reed S. I. (2017). “Cyclin E Deregulation and Genomic Instability,” in DNA Replication. Editors Masai H. Foiani M. (Singapure: Springer), 527–547. 10.1007/978-981-10-6955-0_22 29357072

[B111] Teixeira L. K. Wang X. Li Y. Ekholm-Reed S. Wu X. Wang P. (2015). Cyclin E Deregulation Promotes Loss of Specific Genomic Regions. Curr. Biol. 25, 1327–1333. 10.1016/j.cub.2015.03.022 25959964 PMC4439338

[B112] Thompson L. L. Baergen A. K. Lichtensztejn Z. McManus K. J. (2020). Reduced SKP1 Expression Induces Chromosome Instability through Aberrant Cyclin E1 Protein Turnover. Cancers 12, 531. 10.3390/cancers12030531 PMC7139525 32106628

[B113] Tokuyama Y. Horn H. F. Kawamura K. Tarapore P. Fukasawa K. (2001). Specific Phosphorylation of Nucleophosmin on Thr199 by Cyclin- Dependent Kinase 2-Cyclin E and its Role in Centrosome Duplication. J. Biol. Chem. 276, 21529–21537. 10.1074/jbc.M100014200 11278991

[B114] Toledo L. I. Murga M. Zur R. Soria R. Rodriguez A. Martinez S. (2011). A Cell-Based Screen Identifies ATR Inhibitors with Synthetic Lethal Properties for Cancer-Associated Mutations. Nat. Struct. Mol. Biol. 18, 721–727. 10.1038/nsmb.2076 21552262 PMC4869831

[B115] Welcker M. Singer J. Loeb K. R. Grim J. Bloecher A. Gurien-West M. (2003). Multisite Phosphorylation by Cdk2 and GSK3 Controls Cyclin E Degradation. Mol. Cell 12, 381–392. 10.1016/s1097-2765(03)00287-9 14536078

[B116] Westendorp B. Mokry M. Groot Koerkamp M. J. A. Holstege F. C. P. Cuppen E. de Bruin A. (2012). E2F7 Represses a Network of Oscillating Cell Cycle Genes to Control S-phase Progression. Nucleic Acids Res. 40, 3511–3523. 10.1093/nar/gkr1203 22180533 PMC3333892

[B117] Won K. A. Reed S. I. (1996). Activation of Cyclin E/CDK2 Is Coupled to Site-specific Autophosphorylation and Ubiquitin-dependent Degradation of Cyclin E. EMBO J. 15, 4182–4193. 10.1002/j.1460-2075.1996.tb00793.x 8861947 PMC452142

[B118] Ye X. Nalepa G. Welcker M. Kessler B. M. Spooner E. Qin J. (2004). Recognition of Phosphodegron Motifs in Human Cyclin E by the SCFFbw7 Ubiquitin Ligase. J. Biol. Chem. 279, 50110–50119. 10.1074/jbc.M409226200 15364936

[B119] Zack T. I. Schumacher S. E. Carter S. L. Cherniack A. D. Saksena G. Tabak B. (2013). Pan-cancer Patterns of Somatic Copy Number Alteration. Nat. Genet. 45, 1134–1140. 10.1038/ng.2760 24071852 PMC3966983

[B120] Zariwala M. Liu J. Xiong Y. (1998). Cyclin E2, a Novel Human G1 Cyclin and Activating Partner of CDK2 and CDK3, Is Induced by Viral Oncoproteins. Oncogene 17, 2787–2798. 10.1038/sj.onc.1202505 9840943

[B121] Zegerman P. Diffley J. F. X. (2007). Phosphorylation of Sld2 and Sld3 by Cyclin-dependent Kinases Promotes DNA Replication in Budding Yeast. Nature 445, 281–285. 10.1038/nature05432 17167417

[B122] Zhao H. Chen X. Gurian-West M. Roberts J. M. (2012). Loss of Cyclin-dependent Kinase 2 (CDK2) Inhibitory Phosphorylation in a CDK2AF Knock-In Mouse Causes Misregulation of DNA Replication and Centrosome Duplication. Mol. Cell. Biol. 32, 1421–1432. 10.1128/MCB.06721-11 22331465 PMC3318579

[B123] Zhao J. Kennedy B. K. Lawrence B. D. Barbie D. A. Matera A. G. Fletcher J. A. (2000). NPAT Links Cyclin E-Cdk2 to the Regulation of Replication-dependent Histone Gene Transcription. Genes Dev. 14, 2283–2297. 10.1101/gad.827700 10995386 PMC316937

